# Transformer encoder with multiscale deep learning for pain classification using physiological signals

**DOI:** 10.3389/fphys.2023.1294577

**Published:** 2023-12-06

**Authors:** Zhenyuan Lu, Burcu Ozek, Sagar Kamarthi

**Affiliations:** Department of Mechanical and Industrial Engineering, Northeastern University, Boston, MA, United States

**Keywords:** pain intensity classification, multiscale convolutional networks, transformer encoder, squeeze-and-excitation residual network, deep learning, EDA, temporal convolutional network, BioVid

## Abstract

Pain, a pervasive global health concern, affects a large segment of population worldwide. Accurate pain assessment remains a challenge due to the limitations of conventional self-report scales, which often yield inconsistent results and are susceptible to bias. Recognizing this gap, our study introduces PainAttnNet, a novel deep-learning model designed for precise pain intensity classification using physiological signals. We investigate whether PainAttnNet would outperform existing models in capturing temporal dependencies. The model integrates multiscale convolutional networks, squeeze-and-excitation residual networks, and a transformer encoder block. This integration is pivotal for extracting robust features across multiple time windows, emphasizing feature interdependencies, and enhancing temporal dependency analysis. Evaluation of PainAttnNet on the BioVid heat pain dataset confirm the model’s superior performance over the existing models. The results establish PainAttnNet as a promising tool for automating and refining pain assessments. Our research not only introduces a novel computational approach but also sets the stage for more individualized and accurate pain assessment and management in the future.

## 1 Introduction

An estimated 25.3 million adults reportedly have experienced daily pain for the last 3 months in the U.S., and almost 40 million adults suffer from severe pain, leading to deteriorating health conditions (Nahin, 2015). Building on this, recent studies show that individuals enduring chronic pain are five times more likely to be afflicted with mental disorders such as depression or anxiety compared to those without chronic pain (De La Rosa et al., 2023). Furthermore, the prevalence of chronic pain outnumbers other prevalent chronic conditions like diabetes and hypertension, with an annual incidence rate of 52.4 cases per 1,000 (Nahin et al., 2023).

Pain serves as a multi-faceted biological alarm system, indicating potential or ongoing tissue damage, defined by Merskey *et al.* (Merskey, 1979). This alarm system is not merely physiological but also engages psychological and emotional dimensions. Its primary function is to activate the body’s defense mechanisms, aiming to counteract harmful stimuli and mitigate further tissue damage.

Over the last 2 decades, the field of pain research has seen significant growth, both in terms of interest and scholarly output. A comprehensive review study by Ozek *et al.* analyzed 264,560 scientific articles published from 2002 and reveals the growth in pain research. A sevenfold increase in the use of ‘pain’ as a keyword nearly tripled the number of research papers discussing pain (Ozek et al., 2023). Particularly, they have been focusing on topics such as chronic pain, pain management, pain assessment, and neuropathic pain. Recent trends between 2017 and 2021 indicate a multidisciplinary approach, exploring pain’s relationship and management with opioids, analgesia, and psychological factors such as anxiety and quality of life.

Despite these advancements in understanding pain and pain management are noteworthy, a significant gap exists in the area of accurate and objective pain assessment (Hämäläinen et al., 2022). Accurate pain assessments are critical for monitoring the effectiveness of pain management strategies and observing changes in pain severity over time. These assessments are particularly crucial in clinical settings, where they guide healthcare providers in customizing treatment plans (Leigheb et al., 2017).

A key aspect of these assessments lies in the quantification of pain, often accomplished by measuring its intensity. Widely recognized methods for this purpose include self-report scales such as Visual Analog Scales, Verbal Rating Scales, and Numeric Rating Scales (Lazaridou et al., 2018). While these methods are useful but also have limitations, especially for specific populations such as neonatal infants (Cascella et al., 2019; Eriksson and Campbell-Yeo, 2019) and individuals with cognitive impairments or communication barriers (Deldar et al., 2018; Werner et al., 2022). This limitation underscores the need for more automated and objective techniques (Zamzmi et al., 2018).

Physiological signals, including electrodermal activity (EDA), electrocardiography (ECG), electromyography (EMG), and electroencephalography (EEG), are frequently employed for pain intensity classification (Werner et al., 2022). Among these, EDA, also known as galvanic skin response (GSR), has garnered particular interest for its non-invasive nature and ease of data acquisition through wearable sensors (Chen et al., 2021a). EDA measures variations in skin conductance, serving as a valuable indicator of pain (Ledowski et al., 2009; Braithwaite et al., 2013). Its ease of data collection and the insights it provides into the body’s physiological response to pain make it a practical choice for real-time and continuous monitoring (Erekat et al., 2021). However, traditional methods often fall short of capturing the complexities inherent, especially the temporal features, in EDA responses to pain.

Recognizing this limitation, our study introduces a deep learning framework, PainAttnNet, conceived to classify pain levels using physiological signals. PainAttnNet is an innovative model integrating Multiscale Convolutional Network (MSCN), a Squeeze-and-Excitation Residual Network (SEResNet), and a transformer encoder block.

1) The MSCN is designed to extract both short-, medium- and long-window sequential features from signals. The architecture can capture essential information about the overall trends and variations in the physiological data, offering valuable insight into the pain intensity.

2) The SEResNet in proposed model learns the interdependencies among the extracted features, enhancing their representation capability. This network selectively weights the importance of different channels and adaptively recalibrates the feature maps, thereby improving the model’s sensitivity to the most informative features.

3) The transformer encoder block in PainAttnNet extracts the temporal representations. This block uses a multihead attention layer in conjunction with a temporal (causal) convolutional network, allowing the network to process the input sequence simultaneously, while effectively capturing the dependencies between the input and output over time.

Our contributions are twofold. First, we introduce a deep learning framework with multiple modules adopted from different fields and previous studies, which effectively classifies pain intensity from physiological signals by utilizing various strategies to capture the features. Second, we demonstrate that PainAttnNet outperforms the existing models in classifying pain intensities, indicating its potential for automated pain intensity classification.

## 2 Related work

### 2.1 Pain classification

#### 2.1.1 Conventional machine learning models

Conventional machine learning models have served as foundational parts in the domain of pain intensity classification. Models such as k-Nearest Neighbors (KNN) were explored by Cao *et al*. (Cao et al., 2021), while the Support Vector Machine (SVM) approach was researched by Campbell *et al*. (Campbell et al., 2019). Bayesian models have also found their place in this domain, with notable work by Santra *et al*. (Santra et al., 2020). Tree-based models, especially XGBoost and AdaBoost, have been frequently utilized, with research by Shi *et al*. (Shi et al., 2022), Naeini *et al*. (Naeini et al., 2021), and Cao *et al*. (Cao et al., 2021) leading the way. A notable combination was by Pouromran *et al*., who integrated BiLSTM with XGBoost for more nuanced pain intensity classification (Pouromran et al., 2022).

#### 2.1.2 MLP-based models

Multilayer perceptron (MLP), being feedforward neural networks, have been commonly used in the domain of pain intensity classification. Lopez-Martinez and Picard (Lopez-Martinez and Picard, 2017) introduced a deep MLP model tailored for classifying pain intensity based on physiological signals. Gouverneur *et al*. (Gouverneur et al., 2021) further applied MLP, emphasizing its utility when combined with distinct hand-crafted features for classifying heat-induced pain.

#### 2.1.3 RNN-based models

Recurrent Neural Networks (RNNs), especially their advanced variants, have been recognized for their capacity to handle time-series data, making them particularly apt for pain signal classification. The BiLSTM model, an evolution of the traditional RNN, addresses challenges like vanishing and exploding gradients. A notable application was presented by Wang *et al*. (Wang et al., 2020), who integrated a BiLSTM layer for temporal feature extraction, further enhanced with hand-crafted features. Then the features are sent to a MLP block for classification. Furthermore, Pouromran *et al*. (Pouromran et al., 2022) demonstrated an innovative combination by employing BiLSTM for feature extraction, which was then processed by XGBoost, providing a nuanced approach to pain intensity classification.

#### 2.1.4 CNN-based models

Convolutional Neural Network (CNN) models have significantly transformed pain analysis by offering both recognition and classification capabilities. Thiam *et al.* (Thiam et al., 2019) proposed a model using a deep Convolutional Neural Network (CNN) framework followed by fully connected layers (FCL). This model was primarily tailored for pain recognition, and then leveraging spatial features from data for accurate binary classification: ‘pain’ or ‘no pain’. With modifications and given suitable training data, such architectures have the inherent potential for broader classification tasks, such as categorizing different pain levels or types. Similarly, Subramaniam and Dass (Subramaniam and Dass, 2020) developed a hybrid deep learning model that combines the strengths of CNN, for spatial feature extraction, with LSTM to capture temporal dynamics. The extracted features were then processed by an FCL to categorize the signals into ‘pain’ or ‘no pain’ categories.

#### 2.1.5 Limitations

While these models have shown potential in classifying pain intensities, they possess inherent limitations. RNNs, despite their capacity for capturing temporal dependencies in sequential data, can struggle with long-term dependencies in the input sequences and their sequential nature hampers parallel training. MLPs, on the other hand, may not effectively capture temporal dependencies in input signals. CNNs have been shown as a powerful tool in the domain of pain intensity classification due to their capability in spatial feature extraction from data. Their ability to identify patterns in the data that are crucial for pain recognition. However, when it comes to EDA data, which is inherently time-series in nature, CNNs might face challenges. Specifically, traditional CNN architectures, while effective for many tasks, may not be optimally designed to capture these temporal dependencies in EDA signals, underscoring the need for hybrid models that can better handle time-series data. To overcome these limitations, we introduce PainAttnNet, a framework leveraging a transformer encoder for pain intensity classification using physiological signals.

### 2.2 Feature extraction

CNN has proven its efficacy in various tasks, *e.g.*, audio classification (Lee et al., 2009) and image classification (Krizhevsky et al., 2017). Nevertheless, traditional CNNs operate at a fixed scale, extracting features at one level of granularity. This can result in overlooking significant features that exist across multiple scales or frequencies. In response to this limitation, Multiscale Convolutional Neural Network (MSCN) were developed. MSCN has a unique multiscale layer and learnable convolutional layers, enabling the automatic extraction of features at diverse scales and frequencies. This capacity allows MSCN to discern more intricate patterns in the data that may be overlooked by conventional CNNs, potentially leading to superior feature representation and enhanced classification performance (Cui et al., 2016; Li and Yu, 2016). Fu *et al.* (Fu et al., 2018) introduced a novel architecture to overcome the limitations of depth estimations by incorporating multi-scale information concatenated channel-wise. In a similar vein, Gong *et al.* (Gong et al., 2019) extracted deep multiscale features from hyperspectral images, thereby improving the model’s performance. Moreover, Peng *et al.* (Peng et al., 2020) integrated traditional signal filtering techniques with CNNs to develop a multiscale network for feature extraction to diagnose wheelset-bearing faults under strong noise and variable load conditions. These applications demonstrate the potential of MSCNs to discern more complex patterns in the data, leading to superior feature representation and improved classification performance. We adopt MSCN in PainAttnNet to effectively capture intricate, multi-scale patterns in physiological signals, thus enhancing pain intensity classification. The features extracted at different scales are merged via channel-wise concatenation, which preserves unique information and provides a robust, comprehensive feature representation.

Hu *et al.* (Hu et al., 2018) introduced the Squeeze-and-Excitation Network (SENet). It has gained attention as a critical tool for efficient feature extraction and representation. SENet enhances the network’s representational power by modeling interdependencies between convolutional feature map channels. Its utility is showcased in various applications such as EEG seizure detection by Li *et al.* (Li et al., 2020), sleep staging based on multi-modal physiological signals by Jia *et al.* (Jia et al., 2022), and single EEG channel sleep classification where SENet was applied for feature extraction by Eldele *et al.* (Eldele et al., 2021), demonstrating superior performance. Building on the success of SENet, the Convolutional Block Attention Module (CBAM) expands the concept by refining feature maps along both spatial and channel dimensions. However, the increased complexity associated with CBAM can be a double-edged sword, enhancing performance at the cost of increased computational demands (Woo et al., 2018). In this context, the simplicity and effectiveness of Residual Networks (ResNets) provide a significant benefit. ResNets tackle the vanishing gradient problem and provide a supportive structure for SENet, enabling optimal utilization of all channels in the feature maps (He et al., 2016). The combination of ResNets and SENet capitalizes on the strengths of both, making it a crucial component of PainAttnNet. This combination highlights the potential of incorporating these robust networks into our framework, thereby enhancing its performance in pain intensity classification tasks.

Temporal Convolutional Networks (TCNs) have found successful applications across a range of domains. In the domain of action segmentation, TCNs have been utilized as an effective method for action segmentation. These networks have shown superior ability in capturing long-range relationships, longer segment durations, and complex action compositions compared to LSTM (Lea et al., 2016). In audio processing, TCNs have been utilized for generating raw audio waveforms, achieving state-of-the-art performance in musical audio synthesis and text-to-speech tasks. This achievement underscores the ability of TCNs to model complex patterns in temporal data (Oord et al., 2016). Van Den Oord *et al.* (Van den Oord et al., 2016) presented a model that conditions PixelCNN on latent space for specific image class generation. This innovative approach showcases the potential of merging TCNs with architectures like PixelCNN. In proposed model, we employ TCNs to extract temporal representations from physiological signals. These representations are then sent to a Transformer Encoder, an architecture built on the attention mechanisms first introduced by Bahdanau *et al.* (Bahdanau et al., 2014) and later refined by Vaswani *et al.* (Vaswani et al., 2017), for effective handling of sequence dependencies.

The Transformer architecture revolutionized the machine learning field by proposing a model that relies solely on self-attention mechanisms, thereby discarding the need for recurrent layers. Dosovitskiy *et al.* (Dosovitskiy et al., 2020) demonstrated the effectiveness of Transformer Encoders in computer vision field, showing their ability to outperform CNNs in image recognition tasks when trained on large-scale datasets. Another work introduced a dual-branch transformer that combines image patches to generate better image representations, demonstrating the potential of Transformer Encoders in handling multi-scale data (Chen et al., 2021b). In proposed model, we utilize the Transformer Encoder to further enhance the extraction of temporal features, thereby improving the classification of pain intensity from physiological signals.

## 3 Methodology

**FIGURE 1:**
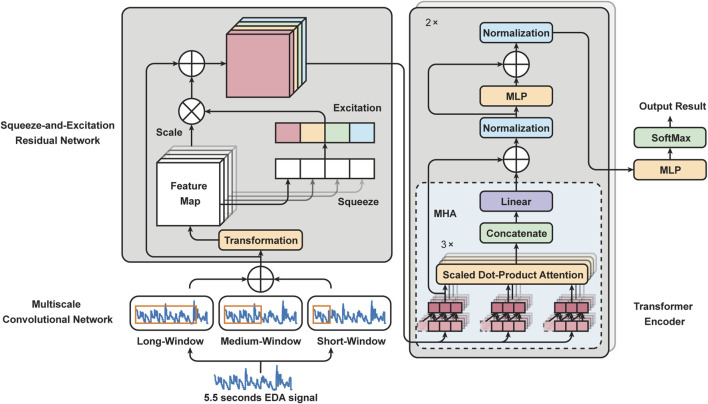
Outline framework of our proposed PainAttnNet. Left bottom: Multiscale Convolutional Network (MSCN). Left top: Squeeze-and-Excitation Residual Network (SEResNet). Right: Transformer Encoder.

Building on the importance of EDA in pain intensity classification, as highlighted in the introduction, we introduce a novel framework for automated pain assessment named PainAttnNet. The architecture of this framework is outlined in Figure 1. This framework 1) applies a multiscale convolutional network (MSCN) to extract multiscale features from EDA. 2) Following this, we incorporate a Squeeze-and-Excitation Residual Network (SEResNet) to boost the interpretability of the extracted features by understanding their interdependencies. 3) A multi-head attention framework combined with a TCN is used to encapsulate the temporal aspects of the extracted features. Supplemental information and source code are available at: https://github.com/zhenyuanlu/PainAttnNet.

### 3.1 Multiscale convolutional network (MSCN)

**FIGURE 2:**
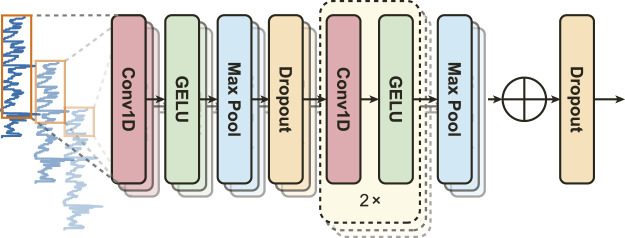
The structure of the multiscale convolutional network (MSCN).

EDA signals are inherently non-stationary, necessitating a model capable of capturing diverse features. PainAttnNet approach employs a Multiscale Convolutional Network (MSCN) designed to sample varied lengths of EDA signal sequences through three convolutional layers (Figure 2). Taking inspiration from deep learning models from several studies (Li and Yu, 2016; Gong et al., 2019; Peng et al., 2020; Eldele et al., 2021), the branches cover windows of 2 s, 1 s, and 0.1 s using kernels of 1,024, 512, and 50, respectively.

The MSCN architecture, depicted in Figure 2, includes two max-pooling layers and three convolutions per branch. The output from each convolution is normalized by a batch normalization block before the Gaussian Error Linear Unit (GELU). Max-pooling, a downsampling technique, reduces feature map dimensionality and controls overfitting by determining the maximum value in a given feature map region. Consider an input $X=\left\{x_{1},\ldots ,x_{N}\right\}\in R^{N\times L\times C}$ , the max-pooling operation can be represented as:

$$f_{c}\left(x\right)=\underset{i,j}{\max}\left(x_{i,j,c}\right).$$ (1)

here, *f* represents the feature map, **x** denotes the input feature map for each channel, *c* denotes the channel, *i* and *j* are the dimensions. The max pooling operation is used for each channel separately. The function *f*_*c*_(**x**) outputs the maximum value among the elements present in channel *c*. For instance, *f*_*c*_(**x**) in the feature map **X** corresponds to the largest value of all elements residing at the *c*th channel.

After each convolutional layer, the batch normalization layer accelerates network convergence by decreasing internal covariate shifts and stabilizes the training process (Ioffe and Szegedy, 2015). Batch normalization normalizes the activations of the previous network by using channel-wise mean *μ*_*c*_ and standard deviation *σ*_*c*_. The batch normalization formulas are as follows: Let feature map $X\in R^{N\times L\times C}$ over a batch, where *C* is the channel, *L* represents the length of each feature, and *N* denotes the overall number of features. The formula for batch normalization is as follows:

$$y_{\gamma ,\beta ,c}=\frac{x_{i,j,c}-\mu_{c}}{\sigma_{c}}\cdot \gamma +\beta ,$$ (2)

here,

$$\mu_{c}=\frac{1}{NL}\underset{i,j}{\sum }x_{i,j,c},$$ (3)

$$\sigma_{c}^{2}=\frac{1}{NL}\underset{i,j}{\sum }{\left(x_{i,j,c}-\mu_{c}\right)}^{2}.$$ (4)

where *c* is the channel index, *i* and *j* are spatial indices; *μ*_*c*_ and $\sigma_{c}^{2}$ are the mean of the values and the variance in channel *c* for the current batch, respectively. In the above equations, *γ* and *β* are learnable parameters introduced to allow the network to learn an appropriate normalization even when the input is not normally distributed.

GELU is a form of activation function that is a smooth approximation of the behavior of the rectified linear unit (ReLU) (Nair and Hinton, 2010) to prevent neurons from vanishing while limiting how deep into the negative regime activations (Hendrycks and Gimpel, 2016). This allows some negative weights to pass through the network, which is important to send the information to the subsequent task in SEResNet. As GELU follows the Batch Normalization Layer, the feature map inputs X∼N(0,1) . The GELU is defined as:

$$g\left(x\right)≔x\cdot \Phi \left(x\right)=x\cdot \frac{1}{2}\left(1+erf\left(\frac{x}{\sqrt{2}}\right)\right).$$ (5)

Here, Φ(**x**) denotes the cumulative distribution function, represented by $P\left(X\leq x\right)$ , and **erf** (⋅) corresponds to the error function. GELU can boost the representation capabilities of the network by introducing a stochastic component that enables more diversity. In addition, it has been demonstrated that GELU has a more stable gradient and a more robust optimization landscape than ReLU and leaky ReLU, because of this GELU can promote faster convergence and improved generalization performance.

Additionally, we employ a dropout layer after the first max pooling in all branches and concatenate the output features channel-wise from these branches of the MSCN.

### 3.2 Squeeze-and-excitation residual network (SEResNet)

**FIGURE 3:**
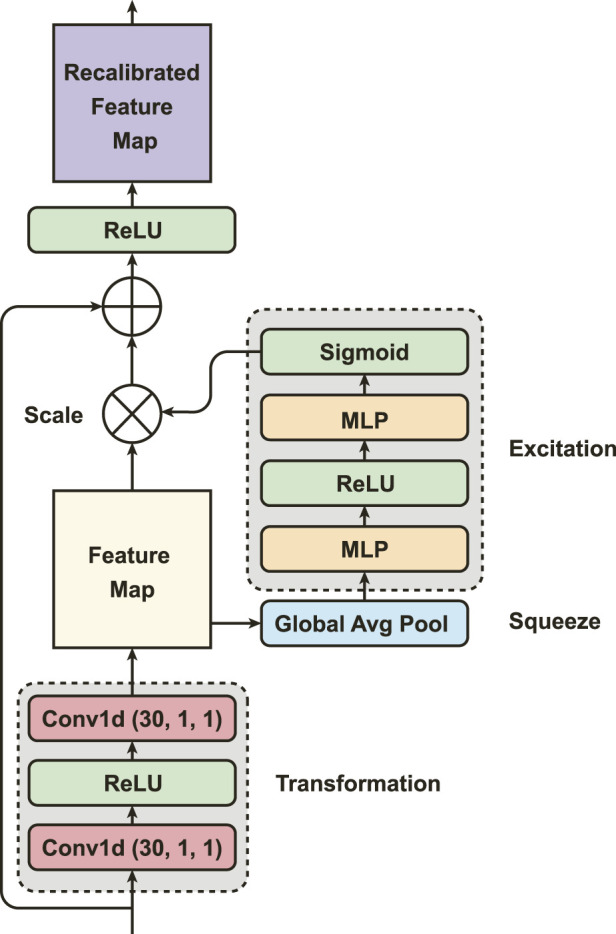
The outline of Squeeze-and-Excitation Residual Network (SEResNet).

Using the SEResNet (Figure 3), we can adaptively recalibrate the concatenated features from the MSCN to enhance the most important global spatial information of EDA signals. The mechanism of the SEResNet aims to model the interdependencies between the channels to enhance the extracted convolutional features and amplify the network’s sensitivity to the most meaningful features (Hu et al., 2018). This recalibration process emphasizes informative features while suppressing less relevant ones, yielding a more interpretable feature representation for subsequent tasks. The SEResNet functions by condensing the channel-wise data of the feature maps into a global information representation, and the excitation operation uses this descriptor to adaptively scale the feature maps (Figure 3).

Particularly, PainAttnNet model starts with the implementation of two convolutions, each having 1 kernel size and 1 stride, with activation conducted via ReLU. Here we use ReLU, other than GELU, to improve the performance on the convergence. At the squeezing stage in the SEResNet, the global spatial information from the two convolutions is then compressed by global average pooling. It reduces the spatial dimensions while keeping the informative features. Let the feature map from the MSCN as $X\in R^{N\times L\times C}$ , we apply two convolutional layers to **X** that results in obtaining new feature maps $V\in R^{N\times L\times C}$ , and then shrink the **V** to generate the statistics $z\in R^{C}$ :

$$z_{c}=\frac{1}{NL}\overset{N}{\underset{i=1}{\sum }}\overset{L}{\underset{j=1}{\sum }}v_{i,j,c},$$ (6)

where **z**_*c*_ is the global average of *L* data points per each channel. Next comes the excitation (adaptive recalibration) stage, in which two FCL generate the statistics used to scale the feature maps. As a bottleneck, the first FCL with ReLU is to reduce the dimensionality. The second FCL with sigmoid recovers the channel dimensions to their original size by performing a dimensionality-increasing operation. Let the $z\in R^{C}$ . We define adaptive recalibration as follows:

$$\alpha =\sigma \left(W_{2}\delta \left(W_{1}z\right)\right),$$ (7)

where *δ* denotes the ReLU, and *σ* represents the sigmoid function. $W_{1}\in R^{\frac{C}{r}\times C}\;\text{and}\;W_{2}\in R^{C\times \frac{C}{r}}$ is the learnable weights for the first and the second FC layer, respectively. Here, *r* is the ratio of reduction. These weights reveal the interdependencies among the channels and provide insights into the most informative channel.

Following this, the original feature map denoted by **v** scaled by the activation *α*, and this is done by channel-wise multiplication:

$$M=\alpha_{c}⊗v_{c},$$ (8)

$$\tilde{X}=X⊕M.$$ (9)

where $\tilde{X}$ is the final output of the SEResNet, which results from the original input **X** and the enhanced features **M**.

### 3.3 Transformer encoder

#### 3.3.1 Temporal convolutional network (TCN)

TCN framework, inspired by the works of Lea *et al.* (Lea et al., 2016) and Van den Oord *et al.* (Oord et al., 2016; Van den Oord et al., 2016), has been used effectively for processing and generating sequential data, *e.g.*, audio or images. TCN employs one-dimensional convolutional layers to extract the temporal dependencies over the sequential input data, like the recalibrated features from SEResNet. In contrast to a regular convolutional network, the output of TCN at a given time *t* depends only on the inputs at times preceding *t*. TCN only permits the convolutional layer to look back in time by masking future inputs. Like the regular convolutional network, each convolutional layer contains a kernel with a specific width to extract certain patterns or dependencies in the input data across time before the present *t*. To preserve the same length for the output and input, one additional padding mechanism is appended to the left side of the input to offset the window shift in the input.

Let input feature map $X\in R^{1\times L\times C_{1}}$ , where *L* is the input length and *C*_1_ is the dimension of the input channels. We have kernel $W\in R^{K\times C_{1}\times C_{2}}$ , and the size of padding (K−1)∈R , where *K* is the kernel size, and *C*_2_ is the dimension of the output channels. Then we have the output from TCN as $\varphi \left(\cdot \right)\in R^{1\times L\times C_{2}}$ . This approach can assist us in constructing an effective auto-regressive model that only retrieves temporal information with a particular time frame from the past without cheating by utilizing knowledge about the future.

#### 3.3.2 Multi-head attention (MHA)

**FIGURE 4:**
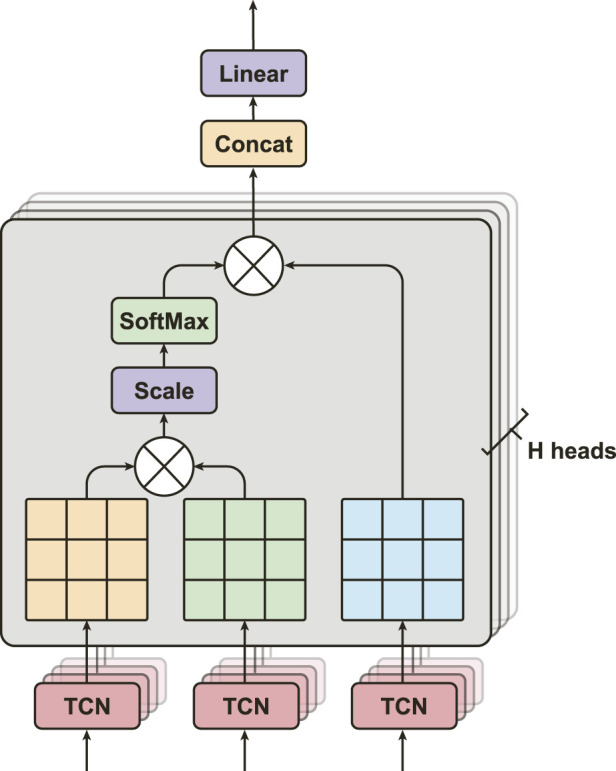
The structure of multi-head attention, consists of H heads of Scaled Dot-Product Attention layers with three inputs from TCNs.

MHA is a popular method for learning long-term relationships in sequences of features (Figure 4). We adapt this algorithm from Vaswani *et al.* (Vaswani et al., 2017), Dosovitskiy *et al.* (Dosovitskiy et al., 2020), and Bahdanau *et al.* (Bahdanau et al., 2014). It has significant performance in different fields, *e.g.*, BERT (Devlin et al., 2019) and GPT (Brown et al., 2020) models in natural language process, and physiological signals classification for sleep Eldele *et al.* (Eldele et al., 2021), Zhu *et al.* (Zhu et al., 2020). MHA consists of multiple layers of Scaled Dot-Product Attention, where each layer is capable of learning different temporal dependencies from the input feature maps (Figure 4). MHA aims to obtain a more comprehensive understanding of how the *i*th feature is relevant to *j*th features by processing them through multiple attention mechanisms. In particular, let the output feature maps from SEResNet, $X=\left\{x_{1},\ldots ,x_{N}\right\}\in R^{N\times L}$ . Then we take three duplicates of **X** such that $\tilde{X}=\varphi \left(X\right)$ , here *φ*(⋅) is the TCN, and $\tilde{X}$ is the output of TCN. Next, we send the three outputs, ${\tilde{X}}^{\left(Q\right)},{\tilde{X}}^{\left(K\right)},{\tilde{X}}^{\left(V\right)}$ to attention layers. This allows us to calculate the weighted sum, the attention scores **z**_*i*_:

$$z_{i}=\overset{L}{\underset{j=1}{\sum }}\alpha_{ij}\varphi \left({\tilde{x}}_{j}^{\left(V\right)}\right),$$ (10)

the weight *α*_*ij*_ of each $\varphi \left({\tilde{x}}_{j}\right)$ is computed by:

$$\alpha_{ij}=\frac{\text{exp}\left(e_{ij}\right)}{\sum_{r=1}^{L}\text{exp}\left(e_{ir}\right)},$$ (11)

here,

$$e_{ij}=\frac{1}{\sqrt{L}}\cdot {\tilde{x}}_{i}^{\left(Q\right)}\cdot {\tilde{x}}_{j}^{\left(K\right)⊤}.$$ (12)

then the output of one attention layer is $z=\left\{z_{0},\ldots ,z_{L}\right\}\in R^{N\times L}$ .

Next, MHA calculates all the attention scores **Z** ^(*H*)^ from multiple attention layers parallelly, and then concatenates them into ${\tilde{Z}}_{\text{MHA}}\in R^{N\times HL}$ , where *H* is the number of attention heads, and *HL* is the overall length of the concatenated attention scores.

We apply a linear transformation with learnable weight $W\in R^{HL\times L}$ to make the input and output sizes the same. This allows us to easily process the subsequent stages. The overall equation for MHA is represented as follows:

$${\tilde{Z}}_{\text{MHA}}=\text{Concat}\left(z^{\left(1\right)},\ldots ,z^{\left(H\right)}\right)\cdot W\in R^{N\times L}.$$ (13)

After concatenating these attention scores, we process them with the original $\tilde{X}$ using an addition operation and layer normalization adopted from (Ba et al., 2016), formed as $\Phi \left(\tilde{X}+{\tilde{Z}}_{\text{MHA}}\right)$ , which can be described as a residual layer with layer norm function *Φ*_1_ (⋅). Then the output of *Φ*_1_ (⋅) is processed by the FCLs and the second residual layer *Φ*_2_ (⋅). Finally, pain intensity categorization results are obtained from two fully connected networks, which are then followed by a Softmax function.

## 4 Experimental results

### 4.1 BioVid Heat Pain Database

**FIGURE 5:**
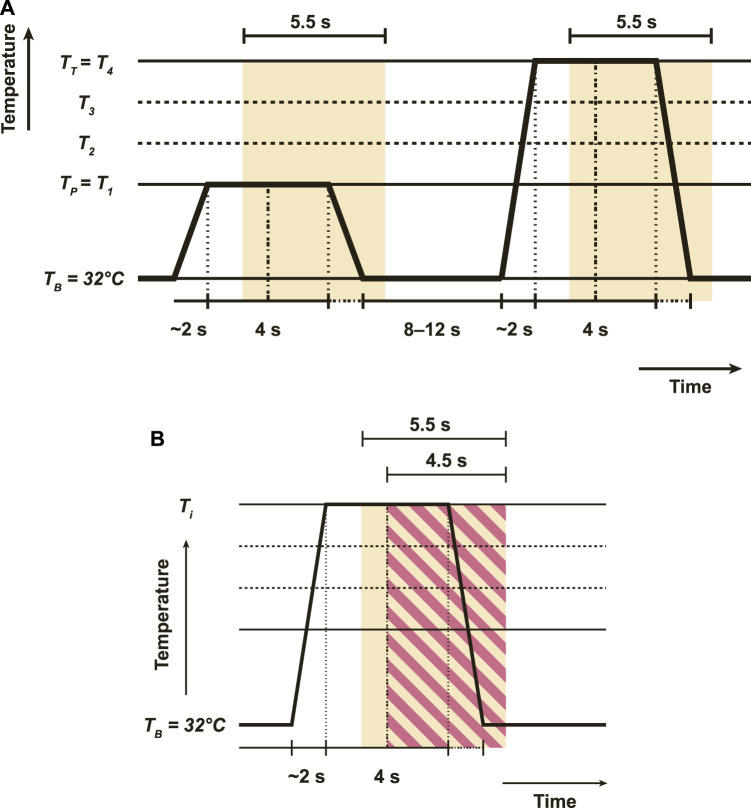
The heat stimuli, with a break in between interval and window segmentations. **(A)** Demonstrates the original experiment settings of BioVid, with a duration of 4 s for each heat stimulus and an interval of 8–12 s between each stimulus. The yellow segmentation displays the 5-s timeframe for each collected signal. **(B)** Thiam *et al.* (Thiam et al., 2019) introduces a different segmentation in red-strip rectangle which takes 4.5 s as opposed to 5.5 s.

In our experiment, we used the Electrodermal Activity (EDA) signals from BioVid Heat Pain Database (BioVid), generated by Walter *et al.* (Walter et al., 2013). As described in Figure 1, Electrodermal Activity (EDA) is a useful indicator of pain intensity (Ledowski et al., 2009). Walter *et al.* (Walter et al., 2013) conducted a series of pain stimulus experiments in order to acquire five distinct datasets, including video signals capturing the subjects’ facial expressions, SCL (also known as EDA), ECG, and EMG. The experiment featured 90 participants in ages: 18–35, 36–50 and 51–65. Each group has 30 subjects, with an equal number of male and female participants. At the beginning of the experiment, the authors calibrated each participant’s pain threshold by progressively raising the temperature from the baseline *T*_0_ = 32°*C* to determine the temperature stages *T*_*P*_ and *T*_*T*_; here *T*_*P*_ represents the temperature stages at which the individual began to experience the heat pain; *T*_*T*_ is the temperature at which the individual experiences intolerable pain. Then four temperature stages can be determined as follows:

$$T_{i}=\left\{\begin{array}{cc} T_{P}+\left[\left(i-1\right)\times \gamma \right]\; & i\in \left\{1,2,3,4\right\}\\ T_{B}\; & i=0 \end{array}\right.$$ (14)

here,

$$\gamma =\left(T_{T}-T_{P}\right)/4$$ (15)

where *T*_*P*_ and *T*_*T*_ are respectively defined as *T*_1_ and *T*_4_. The individual received heat stimuli through a thermode (PATHWAY, Medoc, Israel) connected to the right arm for the duration of the experiment. In each trial, pain stimulation was administered to each participant for a duration of 25 min. In each experiment, they determined five temperatures, *T*_*i*∈{0,1,2,3,4}_, to induce five pain intensity levels from lowest to highest. Each temperature stimulus was delivered 20 times for 4 s, with a random interval of 8–12 s between each application (Figure 5A). During this interval, the temperatures were kept at the pain-free (32°*C*) level. EDA, ECG, and EMG were collected by the according sensors to a sampling rate of 512 Hz with segmentation in a length of 5.5 s. Due to technical issues in the studies, three subjects were excluded, resulting in a final count of 87. Therefore, the training sample of each signal creates a channel with dimensions of 2,816 × 20 × 5 × 87.

Informed by the previous studies (Werner et al., 2014; Lopez-Martinez and Picard, 2017; Gouverneur et al., 2021; Pouromran et al., 2021; Shi et al., 2022; Shi et al., 2022), we adopted the data from BioVid and used the EDA signal in a dimension of 2,816 × 20 × 5 × 87 with a 5.5-s segmentation as the input in our experiment for pain intensity classification based on five pain labels. This 5.5-s window for signal segmentation is the default setting provided by the BioVid database. Our decision to maintain this original 5.5-s window aims to preserve the integrity of the original data, thereby allowing for a comprehensive and unaltered representation of pain signal characteristics. This contrasts with the approach taken by some previous studies. For instance, Subramaniam and Dass *et al.* (Subramaniam and Dass, 2020) removed 20 out of 87 subjects, resulting in 2,816 × 20 × 5 × 67 training samples. In contrast, Thiam *et al.* (Thiam et al., 2019) utilized a 4.5 s segmentation truncating the original time frame by 1 s (Figure 5B). In the next sections, we will compare these latest SOTA methods.

### 4.2 Experimental settings

In our study, we compared PainAttnNet with six baselines, Random Forest (Werner et al., 2014), MT-NN (Lopez-Martinez and Picard, 2017), SVM (Pouromran et al., 2021), TabNet (Shi et al., 2022), MLP (Gouverneur et al., 2021), and XGBoost (Shi et al., 2022). In contrast, we also listed other two models, CNN + LSTM (Subramaniam and Dass, 2020), CNN (Thiam et al., 2019), with different segmentation and sample selections on the EDA signals as the input.

We used 87-fold cross-validation for the BioVid by splitting the subjects into 87 groups, therefore, each subject is in one group as a leave-one-out cross-validation (LOOCV). We trained the model on 86 subjects and tested it on one subject with 100 epochs for each iteration. Ultimately, the macro performance matrices were computed by combining the projected pain intensity classes from all 87 iterations. We created PainAttnNet using Python 3.10 and PyTorch 1.13 on a GPU powered by an Nvidia Quadro RTX 4000. We selected the batch size of 128 for the training dataset, and set the optimizer as Adam applied a weight decay (1e-03) with a 1e-03 initial learning rate. PyTorch’s default settings for Betas and Epsilon were (0.9, 0.999) and 1e-08. In the transformer encoder, we utilized five heads for multi-head attention structure, with each feature’s size being 75.

### 4.3 Performance of PainAttnNet

The performance of PainAttnNet was assessed on the BioVid dataset through five distinctive experimental scenarios: 1) *T*_0_ vs *all* (*T*_1_, *T*_2_, *T*_3_, *T*_4_), 2) *T*_0_ vs *T*_1_, (3)*T*_0_ vs *T*_2_, 4) *T*_0_ vs *T*_3_, and 5) *T*_0_ vs *T*_4_ (refer to Table 1). These tasks were designed to assess the model’s ability to distinguish between various pain intensity levels, with a particular focus on tasks 1, 4, and 5. These tasks are of clinical significance as they involve distinguishing between no pain and various levels of pain intensity, a crucial factor in enhancing patient care.

**TABLE 1:** PainAttnNet’s performance through three evaluation metrics through six tasks: 1) *T*_0_ vs (*T*_1_, *T*_2_, *T*_3_, *T*_4_), 2) *T*_0_ vs *T*_1_, 3) *T*_0_ vs *T*_2_, 4) *T*_0_ vs *T*_3_, and 5) *T*_0_ vs *T*_4_, on BioVid dataset.

Tasks	Specificity	Sensitivity	ACC	*MF*_1_	*κ*
*T*_0_ vs (*T*_1_, *T*_2_, *T*_3_, *T*_4_)	3.05	**99.73**	80.39	47.45	0.04
*T*_0_ vs *T*_1_	43.85	69.54	56.70	55.97	0.13
*T*_0_ vs *T*_2_	67.10	70.48	68.78	68.79	0.38
*T*_0_ vs *T*_3_	81.67	73.16	77.41	77.37	0.55
*T*_0_ vs *T*_4_	**88.28**	82.70	**85.56**	**85.49**	**0.71**

Task 1, *T*_0_ vs *all* (*T*_1_, *T*_2_, *T*_3_, *T*_4_), is a binary classification task that distinguishes between no pain (*T*_0_) and any level of pain all (*T*_1_, *T*_2_, *T*_3_, *T*_4_). Tasks 2 through 5 are binary classification tasks that distinguish between zero pain and each pain level. For instance, Task 2, *T*_0_ vs *T*_1_, aims to differentiate between no pain and low pain.

The performance of PainAttnNet was most impressive on Task 5, achieving an accuracy of 85.56%, a *κ* of 0.71 and an *MF*1 of 85.49%. Conversely, the model’s performance was relatively weaker on Task 1, with an accuracy of 80.39%, a *κ* of 0.04 and an *MF*1 of 47.45%. The performance on Tasks 2, 3, and 4 was intermediate, with varying levels of accuracy, Cohen’s Kappa, and macro F1 score.

We further compared the performance of PainAttnNet with other SOTA models on the BioVid dataset for pain intensity classification (refer to Table 2). For ease of comparison, we selected four of the six classification tasks: *T*_0_ vs *T*_1_, *T*_0_ vs *T*_2_, *T*_0_ vs *T*_3_, and *T*_0_ vs *T*_4_.

**TABLE 2:** The performance comparison between PainAttnNet and other SOTA approaches. CNN + LSTM^‡^ (Subramaniam and Dass, 2020); CNN^‡^ (Thiam et al., 2019); CNN^‡^ (Thiam et al., 2019); Random Forest (Werner et al., 2014); MT-NN (Lopez-Martinez and Picard, 2017); SVM (Pouromran et al., 2021); TabNet, XGBoost (Shi et al., 2022); MLP (Gouverneur et al., 2021). ‡: as these two approaches proposed two different procedures on the data input, we just list them here but are not able to compare them with others.

Method	*T*_0_ vs *T*_1_	*T*_0_ vs *T*_2_	*T*_0_ vs *T*_3_	*T*_0_ vs *T*_4_	Procedure
CNN + LSTM^‡^	85.65	74.47	80.80	80.17	5.5s Segment, *n* = 67 × 20 × 5
CNN^‡^	61.67	66.93	76.38	84.57	4.5s Segment, *n* = 87 × 20 × 5
Random Forest	55.40	60.20	65.90	73.80	5.5s Segment; *n* = 87 × 20 × 5
MT-NN	50.01	60.34	69.76	79.98	
SVM	-	-	-	83.30	
TabNet	**65.57**	67.76	74.54	83.99	
MLP	59.08	65.09	75.14	84.22	
XGBoost	61.49	68.39	76.15	85.23	
PainAttnNet (Ours)	56.70	**68.78**	**77.41**	**85.56**	

The first two approaches, CNN + LSTM (Subramaniam and Dass, 2020) and CNN (Thiam et al., 2019), employed different sample selections and data segmentation strategies, respectively. Hence, their results are listed in Table 2 but are not directly compared with others.

The proposed model, PainAttnNet, outperformed other SOTA models in tasks *T*_0_ vs *T*_3_, and *T*_0_ vs *T*_4_, where it is critical to distinguish between no pain and nearly intolerable pain. However, in task *T*_0_ vs *T*_2_, PainAttnNet achieved lower accuracy compared to the best-performing SOTA model (68.10% vs. 68.39%). In task *T*_0_ vs *T*_1_, the model introduced by Shi *et al*. (Shi et al., 2022) achieved the highest accuracy.

In conclusion, the comparative analysis underscores the potentiality of PainAttnNet, PainAttnNet, as a robust application for classifying pain intensity levels in Electrodermal Activity (EDA) signals. The model’s performance across various tasks, particularly in distinguishing between no pain and severe pain, highlights its potential utility in clinical settings for improved patient care.

### 4.4 ROC curve analysis

We employed ROC curves to assess the capacity of PainAttnNet to classify varying degrees of pain intensity. The area under the ROC curve (AUC) served as an assessment of performance.

**FIGURE 6:**
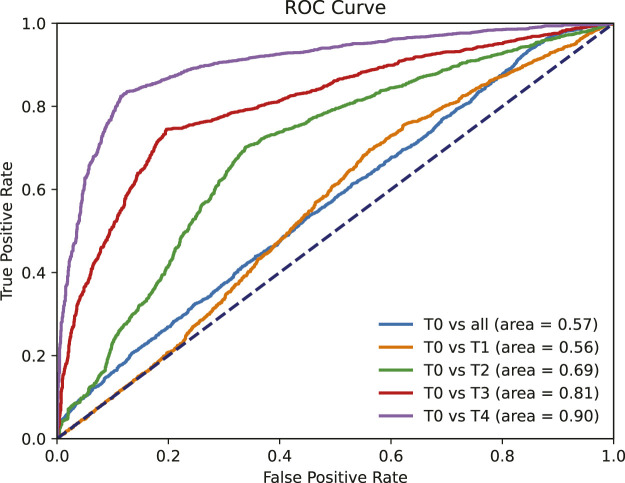
ROC curve for five tasks: *T*_0_ vs *T*_1_, *T*_0_ vs *T*_2_, *T*_0_ vs *T*_3_, *T*_0_ vs *T*_4_, and *T*_0_ vs *all* (*T*_1_, *T*_2_, *T*_3_, *T*_4_).

We conducted ROC curve analyses for five distinct binary classification tasks, yielding AUC scores of 0.56, 0.69, 0.81, 0.9, and 0.57 for the tasks *T*_0_ vs *T*_1_, *T*_0_ vs *T*_2_, *T*_0_ vs *T*_3_, *T*_0_ vs *T*_4_, and *T*_0_ vs *all* (*T*_1_, *T*_2_, *T*_3_, *T*_4_), respectively (Figure 6). PainAttnNet demonstrated a higher proficiency in distinguishing between the absence of pain and high levels of pain than between the absence of pain and lower pain levels.

Despite an accuracy of 80.39%, the AUC for *T*_0_ vs *all* (*T*_1_, *T*_2_, *T*_3_, *T*_4_) was relatively low (0.57). This is due to the model’s low recall for *T*_0_ (3.05%), indicating frequent misclassification of no pain instances as pain, leading to a higher false positive rate and a lower AUC.

The performance of PainAttnNet was superior when distinguishing between the absence of pain and the highest level of pain intensity, which holds considerable practical relevance. However, there is potential for improvement in distinguishing between the absence of pain and lower pain intensities.

In conclusion, PainAttnNet’s performance improves as the difference in pain intensity increases, aligning with recent research and promising for practical applications, especially in distinguishing between no pain and high levels of pain.

### 4.5 Ablation study

In this segment, we elucidate the ablation studies conducted to assess the efficacy of various components in our deep learning model, PainAttNet. The following is our model configuration for the ablation study.

• PainAttNet (Full Model):
  • MSCN (Multiscale Convolutional Neural Network): It provides a method to capture features at various scales and resolutions. This helps in discerning intricate patterns and ensures that features of varying sizes are accounted for in the analysis.
  • SEResNet (Squeeze−and−Excitation Residual Network): It offers an attention mechanism to focus on the most relevant features by dynamically recalibrating channel-wise feature responses. This boosts the model’s sensitivity to important patterns within the data.
  • Transformer Encoder: An architectural paradigm that utilizes self−attention mechanisms to weigh feature importance, allowing the model to focus on critical aspects of the input data while discarding less relevant information.
• MSCN + Transformer Encoder: By integrating MSCN with the Transformer Encoder, this configuration seeks to capitalize on the MSCN’s spatial feature extraction and the Transformer’s ability to capture long-range temporal dependencies in the data.
• MSCN + SEResNet: By fusing the multiscale feature extraction capabilities of MSCN with the channel-wise recalibration offered by SEResNet, this configuration aims to enhance the focus on important features without the self-attention mechanism of the transformer.
• MSCN Only: This module serves as the foundational model, MSCN Only focuses on extracting multi-scaled spatial features from the input data without the additional enhancements provided by the other components.

**FIGURE 7:**
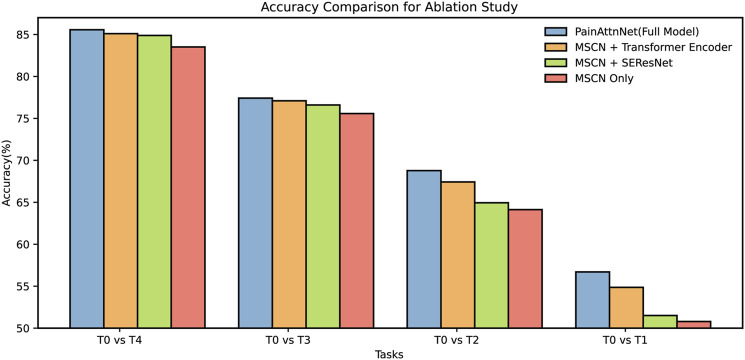
Ablation for four configurations among four tasks: *T*_0_ vs *T*_1_, *T*_0_ vs *T*_2_, *T*_0_ vs *T*_3_, *T*_0_ vs *T*_4_.

Across all tasks, PainAttnNet consistently outperforms other configurations (Figure 7). This reinforces the cumulative advantage of integrating MSCN, SEResNet, and the transformer encoder components into a unified architecture. The accuracy trends observed for MSCN when paired with either the Transformer Encoder or SEResNet suggest their value addition over solely utilizing the MSCN. Both combinations consistently deliver improved results over the MSCN Only configuration across the specified tasks. It is evident that while the accuracy improvements in some configurations may seem marginal, they are nonetheless significant. Even slight increments in accuracy can underscore the capability of the model to capture intricate nuances within the pain signal data, especially when dealing with real-world datasets.

In summation, the ablation study results demonstrate the inherent benefits of these specific architectures, with PainAttnNet manifesting as the most proficient.

### 4.6 Visualization of SE Module Recalibration

**FIGURE 8:**
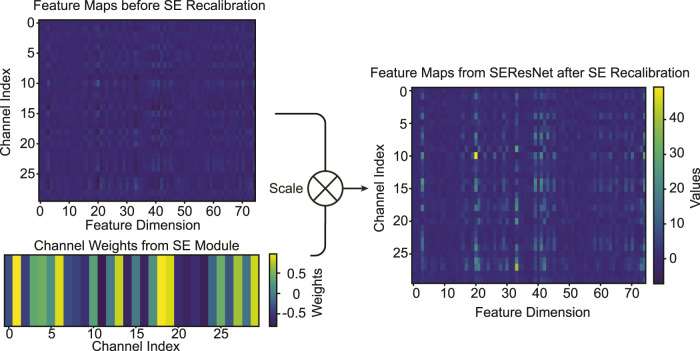
Visualization of average feature maps (*T*_4_) pre and post SE recalibrartion. The bottom-left plot depicts the learned channel weights, guiding the recalibration. The comparison between the top-left and top-right plots illustrates the adaptive feature recalibration effected by the SE module.

The Squeeze-and-Excitation (SE) module’s primary purpose is to adaptively recalibrate channel-wise feature responses. This recalibration emphasizes certain informative features while diminishing less relevant ones, providing a more refined feature representation. The visualizations in Figure 8 are derived from the trained model in the previous sections. To elucidate the SE module’s recalibration effects, we processed the entire training dataset through the trained model. By examining the averaged feature maps from these samples, we intended to highlight the consistent patterns of recalibration that the SE module introduces, both before and after its operation.

Before SE Module Recalibration: As visualized in the top-left plot of Figure 8, the “Feature Maps before SE Recalibration” exhibits the distribution of channel-wise features. This representation is the outcome post the Multiscale Convolutional Network (MSCN) processing. SE Channel Importance Weights: The bottom-left plot of Figure 8 showcases the “Channel Weights for SE Module,” which are learned during the training process. These weights dictate the importance of each channel and subsequently guide the SE operation in recalibrating the features. After SE Module Recalibration: In the top-right plot of Figure 8, one can observe the feature map “after” SE recalibration. Distinct changes in the feature intensity and emphasis are evident, with some features becoming more pronounced, while others diminish. While it is apparent that the SE module emphasizes certain features and diminishes others, identifying the specific nature or type of these features is non-trivial. This is primarily because the features have already been processed by the MSCN, making their innate characteristics intricate to identify purely based on SE module visualization.

### 4.7 Sensitive analysis on MSCN scales

**FIGURE 9:**
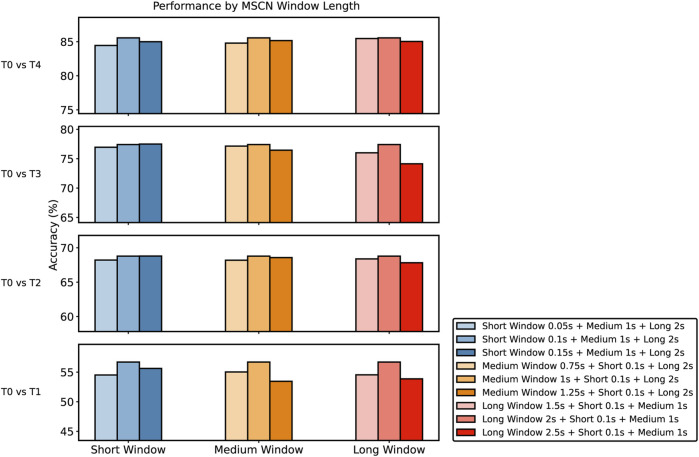
Comparison of the performance of different MSCN Window Lengths against the baseline across *T*_0_ vs *T*_1_, *T*_0_ vs *T*_2_, *T*_0_ vs *T*_3_, and *T*_0_ vs *T*_4_ Task. For each experiment, only one window length was altered while keeping other windows constant to ensure isolated effects of the variation. Baseline settings: short window, 0.1s; medium window, 1s; long window, 2s.

Our analysis investigates three specific window scale combinations (Figure 9): short, medium, and long. The chart in Figure 9 presents the performance variations observed across different MSCN window lengths and four tasks. Each of the bars corresponds to a specific window length combination. Our chosen baseline of Short Window 0.1s + Medium Window 1s + Long Window 2s consistently performs well across various tasks, even if it does not always achieve the highest accuracy. We have thoroughly examined all configurations and found that the baseline performs solidly in most cases, making it the best overall choice. While some combinations slightly outperform the baseline in certain situations, these nuanced differences do not show a consistent improvement, confirming our confidence in our chosen baseline.

The width of the window in the MSCN is a pivotal parameter influencing the model’s performance. Each window length captures specific features from the pain signals, enabling the model to analyze patterns at various temporal granularity. Longer windows provide a broader view, capturing low-frequency components and global patterns, while shorter windows allow the model to capture high-frequency components. The integration of these diverse window lengths enables the model to construct a comprehensive and multi-granular feature representation, enhancing its capacity to discern subtle patterns and thereby improving its overall predictive performance.

## 5 Discussion and conclusion

PainAttnNet, the framework we introduced, serves as a novel approach for classifying pain severity using EDA signals. The model integrates MSCN and SEResNet for robust feature extraction from EDA signals. A TCN and multiple Scaled Dot-Product Attention layers form the multi-head attention architecture, designed to capture temporal dependencies and relationships among input features. Evaluations on the BioVid heat pain dataset confirm the model’s superior performance over existing methods.

While PainAttnNet demonstrates proficiency in distinguishing the absence of pain from various pain intensities, room for improvement remains, especially in differentiating between distinct levels of pain intensity. One primary limitation is the dataset’s distribution shift among subjects, particularly concerning age and gender demographics. Based on our findings and existing studies, pain perception can vary significantly across different age groups (Murray et al., 2021). Gender differences in pain perception have also been reported, adding another layer of complexity to pain assessment (Keogh, 2022). Additionally, the current dependency on lab-controlled data presents a limitation for the model’s applicability in real-world clinical settings.

## 6 Future work

Moreover, expanding the scope of pain classification signals is pivotal for comprehensive understanding and accuracy. While this paper primarily leverages EDA signals, future iterations of PainAttnNet will incorporate a broader range of physiological signals, such as ECG, EMG, *etc.* Integrating multiple signals can offer a comprehensive view of pain assessment, considering the multifaceted nature of pain responses.

To refine PainAttnNet further, we plan to employ masked models and adaptive embedding for enhanced feature extraction. We also intend to explore the application of contrastive learning in conjunction with domain adaptation on large unlabeled datasets and small segments of labeled data (Zhang et al., 2023). These enhancements aim to improve PainAttnNet’s clinical applicability by addressing its limitations, including those related to age- and gender-based pain perception. Future studies will also aim to train and evaluate the model using more ecologically valid, real-world data.

## Data Availability

Publicly available datasets were analyzed in this study. This data can be found here: https://www.nit.ovgu.de/BioVid.html.
